# White matter repair and treatment strategy after intracerebral hemorrhage

**DOI:** 10.1111/cns.13226

**Published:** 2019-10-02

**Authors:** Yi‐Bin Jiang, Kai‐Yan Wei, Xu‐Yang Zhang, Hua Feng, Rong Hu

**Affiliations:** ^1^ Department of Neurosurgery Southwest Hospital Third Military Medical University Chongqing China

**Keywords:** hemorrhage, regeneration, repair, white matter injury

## Abstract

The predilection site of intracerebral hemorrhage (ICH) is in the basal ganglia, which is rich in white matter (WM) fiber bundles, such as cerebrospinal tract in the internal capsule. ICH induced damage to this area can easily lead to severe neurological dysfunction and affects the prognosis and quality of life of patients. At present, the pathophysiological mechanisms of white matter injury (WMI) after ICH have attracted researchers' attention, but studies on the repair and recovery mechanisms and therapy strategies remain rare. In this review, we mainly summarized the WM recovery and treatment strategies after ICH by updating the WMI‐related content by reviewing the latest researches and proposing the bottleneck of the current research.

## 1 INTRODUCTION

Intracerebral hemorrhage (ICH) is a nontraumatic hemorrhage caused by vascular rupture in the brain parenchyma and accounts for 20%‐30% of all stroke cases in Asia.^1^ ICH is the second most common cause of stroke with a high mortality rate of approximately 30%‐40%.^2^ It should be noted that hypertensive ICH accounts for 70%‐80% of all ICH cases.^3^ Because of the anatomical location, basal ganglia ICH accounts for 50%‐70% of all cases of ICH and could easily lead to disability or even death. The area of the internal capsule of the basal ganglia contains plenty of WM fibers, making it vulnerable to the direct pressure from the hematoma and to secondary damage from hematotoxic products, resulting in hemiplegia (part of the corticospinal tract and cortex injury), hemianopsia (central visual radiation injury), sensory deficit (thalamic central radiation damage), and other sequelae. Magnetic resonance imaging (MRI) showed that WM hyperintensity volumes were higher after ICH than after ischemic strokes. Previous studies also showed that more than 77% of patients with ICH had WM injury (WMI).^4^ Previous studies on ICH in the past decades payed more attention to the impact of hemorrhage on neuron,^5^ insufficient attention to the changes in WM after ICH, which may account for, at least in part, the failure of effectiveness of surgical treatment on ICH patients.

In the recent years, WMI after ICH has gained increasing attention, but researchers mainly focused on the pathophysiological mechanism of WMI in animal models. Considering that the WM in rodents accounts for 10%‐20% of the brain volume, whereas that in humans accounts for 50% of the brain volume, WMI in the human brain may play a more important role. Pathophysiological mechanisms regarding WM injury and recovery are important for understanding ICH and formulating treatments. Previous literatures have reviewed the pathological mechanisms and imaging manifestations, but lacking a systematic and comprehensive summary of the mechanisms of WM recovery or repair, as well as therapeutic strategies for ICH‐induced WMI. Therefore, it was necessary to comprehensively review the latest literatures, summarizing the new tactics to enhance WMI recovery and repair, and update the knowledge on WMI to improve the outcome of patients with ICH.

## 2 THE ORGANIZATION AND FUNCTION OF WHITE MATTER

The gray matter is a complex of neuronal bodies and processes, glial cells, and blood vessels, which are used for local signal transmission. The WM contains bundles of myelinated axons and glial cells. Multiple myelin sheaths produced by mature oligodendrocytes tightly wrap the axons. Damage to the myelin sheaths or even lack of axon integrity will lead to lesions of the WM bundles specialized for higher functions, compromising the accurate and high‐speed neural signal transduction.

White matter fiber bundles can be divided into: (a) projection fibers—fibers in the upward and downward fiber tracts, such as the spinocerebellar tract formed after the gracile and cuneate fasciculi in the medulla oblongata pass through the upper and lower cerebellar foot respectively, the spinothalamic tract linking the brainstem to the dorsal thalamus, the reticular structure arising from the pons and medulla oblongata; they form the communication pathway from the cerebral cortex to the subcortical structures, the interbrain, brainstem, and spinal cord; (b) association fibers—connections between different cortical areas in the ipsilateral hemisphere, such as the longitudinal and the uncinate fasciculus; and (c) commissural tracts—link the brain hemispheres, such as the corpus callosum and the transverse distribution of the bundles of WM fiber tracts.

**Figure 1:**
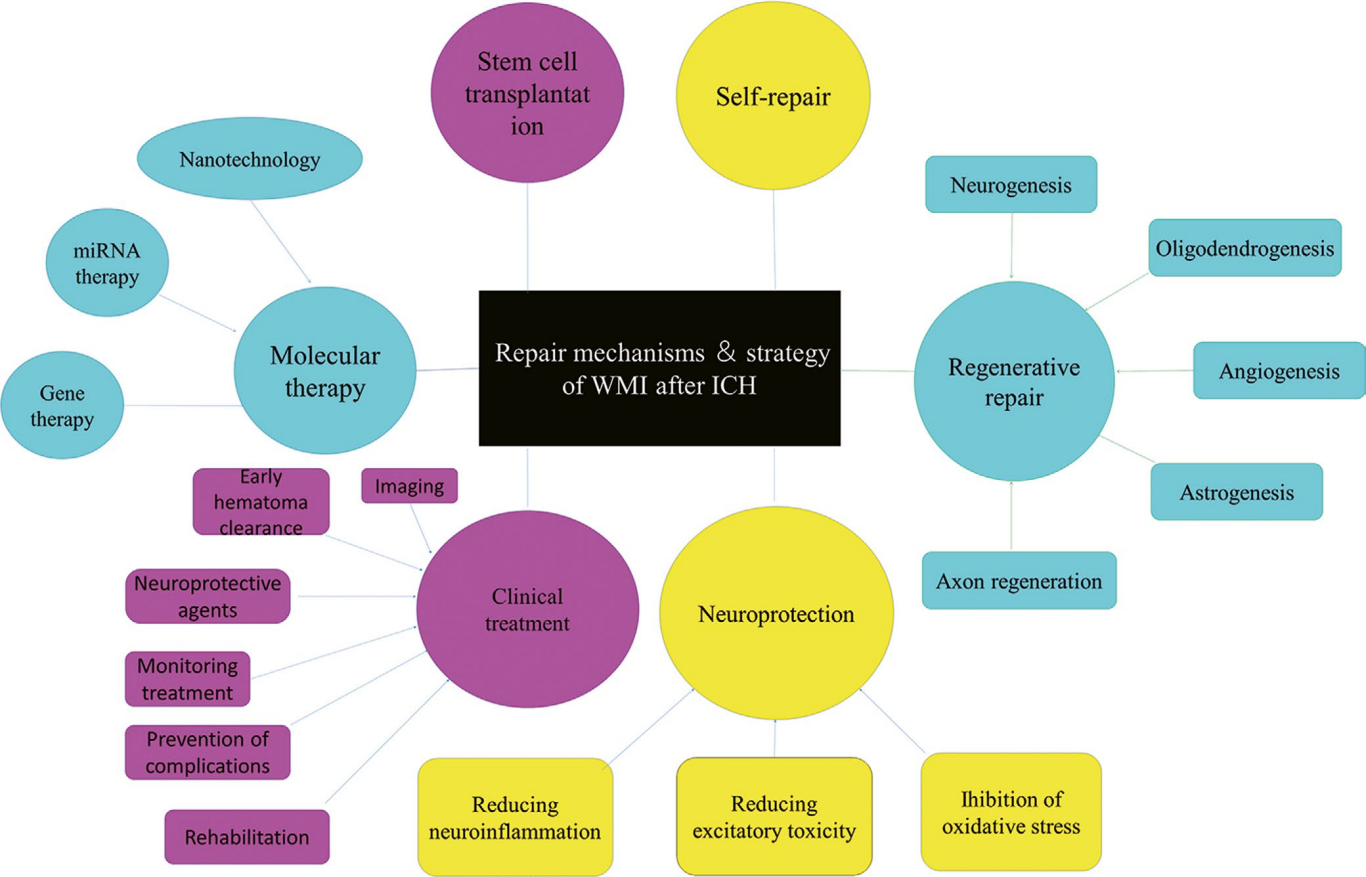
White matter repair mechanism and strategy following ICH. The repair mechanism and strategy of WMI is summarized according to the available literature. The potential therapeutic and current strategies include the following aspects: self‐repair, neuroprotection (reducing neuroinflammation, inhibition of oxidative stress, and reducing excitatory toxicity), regenerative repair (neurogenesis, oligodendrogenesis, angiogenesis, astrogenesis, and axon regeneration), stem cell transplantation, molecular therapy (potential microRNA therapy, gene therapy, and nanotechnology), and clinical treatment (early hematoma clearance, imaging, monitoring treatment, prevention of complications, neuroprotective agents, and early rehabilitation). ICH: intracerebral hemorrhage, WMI: white matter injury

**Figure 2:**
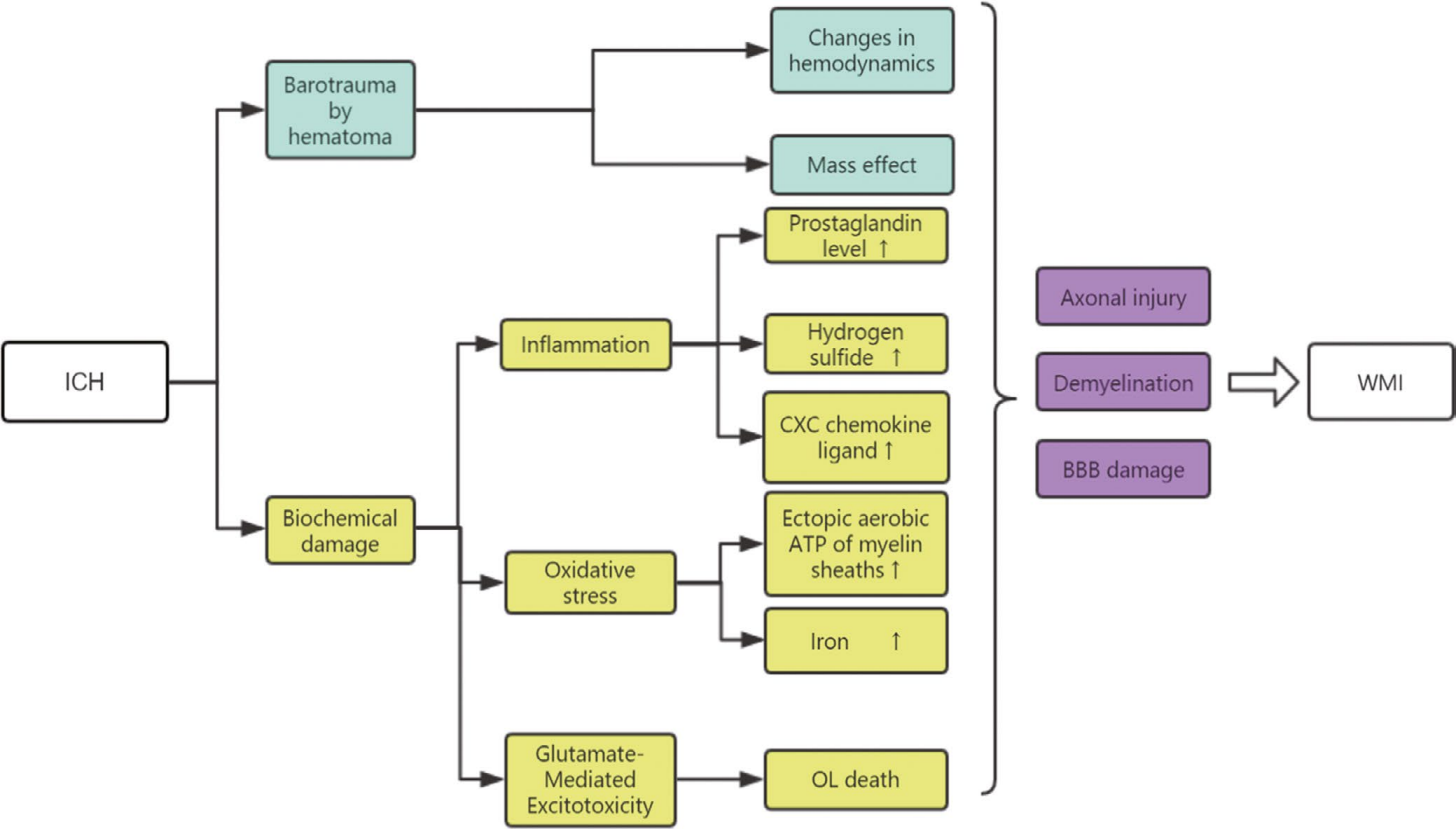
The pathophysiological mechanisms of ICH‐induced WMI were summarized. After ICH, WMI can be caused by primary injury and secondary injury. Primary injury is mainly due to the mass effect, and the changes in hemodynamics caused by the formation of hematoma at the bleeding site after ICH. Secondary injury is mainly induced by neuroinflammation caused by cytokines and chemokines, oxidative stress caused by hemoglobin and its metabolites, as well as neuroexcitatory poisoning mediated by glutamate, which further triggers WMI. Although many of these molecular mechanisms are still being explored, the results are WM axonal damage, demyelination, and blood‐brain barrier destruction. ICH: intracerebral hemorrhage, WMI: white matter injury, OL: oligodendrocyte

Numerous studies have demonstrated that WM structural changes are associated with practicing,^6^, ^7^ studying, reading,^8^ executive control,^9^ cognitive function,^10^, ^11^ type 1 diabetes mellitus,^12^ exercise and speech,^13^ function of the frontal lobe,^14^, ^15^ schizophrenia,^16^ affective disorders,^17^ and even some macro functions, such as breathing.^18^ The WM microstructure is determined by its composition. Previous reviews have summarized the microstructure of the white matter in detail, such as the review by Wang Yuan^19^ and Rosenzweig Shira.^20^ In addition, myelinated axons have a unique molecular structure and organization that enable them to quickly and efficiently transmit the action potentials.^21^ Glial cells in the white matter of the brain have been reported to be implicated in the WMI. Microglia are the inherent immune cells that, when damaged, secrete a variety of factors, such as cytokines and chemokines associated with the axon‐glial damage, contributing to further white matter structural damage.^22^ Glial cells also respond to a variety of extracellular stimuli that cause increased levels of intracellular calcium ions, known as Ca^2+^ signals, which is the basis of glial excitability. Glial calcium signal conduction regulates the release of glial transmitters, such as ATP. ATP binds glial cells in panglial syncytium and exerts effects both on neurons and the cerebrovascular system, regulating the neuronal activity and cerebral blood flow, and playing an important role in the regulating of the glial physiological function and pathology.^23^ ATP‐mediated inositol 1,4,5‐triphosphate (IP3) relies on the Ca^2+^ signaling pathway. Astrocytes and oligodendrocytes express store‐operated calcium entry (SOCE) channels TRPM3 and Orai/Stim, which are essential for glial Ca^2+^ calcium signaling and play a role in the conduction, physiological, and pathological, such as in multiple sclerosis, stroke, and traumatic injury.^24^ A recent clinical study demonstrated that less white matter hyperintensities on MRI were closely related to a greater cerebral vascular density and diameter and a higher cerebral blood flow (Figures 1 and 2).^25^

## 3 PATHOPHYSIOLOGY OF WMI AFTER ICH

Many studies have explored the pathophysiology of WMI after cerebral hemorrhage, including direct compression and barotrauma caused by hematoma, hemodynamic changes caused by ischemia, cerebral edema, destruction of the blood‐brain barrier, effects of red blood cell breakdown products, excitatory toxic effects, oxidative stress, neuroinflammation, and apoptosis.^19^, ^20^, ^26^, ^27^ Many mechanisms about WMI after ICH have been summarized by these above reviews, and recent findings suggest that some molecular signaling pathways involve the process. The elevation of prostaglandin level after cerebral hemorrhage, particularly PEG1, PEG2, and PEG3, can be mediated by interaction with four different G protein‐coupled receptors expressed by neurons and glial cells. It promotes the neuroinflammatory response and induces neuronal apoptosis, leading to further WMI.^28^, ^29^ Zhao et al found that ICH could significantly reduce the production of endogenous hydrogen sulfide (H_2_S) in the brain and that H_2_S weakened the inflammatory damage mediated by NOD‐like receptor (NLR) family pyrin domain‐containing 3 (NLRP3) through the purinergic P2X7 receptor (P2X7R) signaling pathway.^30^ Wang et al reported that microglia also closely interact with the endothelial cells. ICH induced damage to the endothelial cells, resulting in a large number of CXC chemokine ligand (CXCL). ICH sensitized by CXCL induces microglia activation and neutrophil infiltration, leading to WMI caused by neuroinflammation and the blood‐brain barrier (BBB) damage.^31^, ^32^, ^33^ Moreover, antiadhesive matricellular glycoprotein SC1 is a novel early marker of WMI in acute ICH.^34^ There are studies that show that the production of ectopic aerobic ATP of myelin sheaths is related to the oxidative damage of the myelin sheaths, while the oxidative stress induced by ICH can be shown as demyelination. Demyelination reduces the use of lactic acid produced by the axonal myelin and leads to axonal damage due to axon‐glial metabolic coupling disorder (Table 1).^35^, ^36^

**Table 1:** Repair mechanisms and strategy of white matter injury after intracerebral hemorrhage

Repair mechanisms	Repair strategy	Specific treatment
Self‐repair	endogenous clearance of hemoglobin	Upregulation of CD36
	endogenous nerve regeneration	Thrombin improves the function of nerve and vascular regeneration
Neuroprotection	Reducing neuroinflammation	Supplementing P2X7R inhibitor
		Supplementing taurine
		Supplementing curcumin
		Supplementing dimethylamine tetracycline
	Inhibition of oxidative stress	Supplementing ZnPP
		Supplementing baicalein
Regenerative repair	Neurogenesis	transplantation of BMMSCs
		an exogenous lactic acid injection
	Oligodendrogenesis	Supplementing IGF‐1, PDGF, and FGF‐2
		Inhibin A and Matrilin 2
	Angiogenesis	EGb761 attenuates neuronal apoptosis induced by ferrous iron
		Human umbilical cord tissue‐derived cells
		Repeated exposure to low‐intensity ultrasound
		Selective cathepsin B/L inhibitor
	Astrogenesis	—
	Axon regeneration	hydrogels
		ADMSC therapy
		Modulation of GPCR signaling
Stem cell transplantation		Transplantation of BMMSCs, HUCTDCs, and MSCs, and iPSs
Molecular therapy	Potential microRNA therapy	utilizing a unilateral stereotaxic injection to deliver lentiviruses encoding miR‐132, miRNA‐126 or miR‐27b
	Gene therapy	Knocking down IRAK4, CD47
	Nanotechnology	Using peptide‐based nanofiber scaffold
Clinical treatment	Etiological screening	Imaging
	Early hematoma clearance	
	Monitoring treatment	Vital signs
		Blood glucose
		Electrolytes
		Intracranial pressure
		Laboratory parameters
		CT review
	Preventive treatment	Rebleeding
		Edema occupying position
		Seizures
		Pneumonia
		Intracranial infections
	Neuroprotective agents	
	Hypothermia	
	Early rehabilitation	

## 4 IMAGING ANALYSIS OF WMI AFTER ICH

It is difficult to estimate the degree of WMI after ICH because the microstructure of brain tissue is not clearly demonstrated by computed tomography (CT). In recent years, computed tomography perfusion (CTP) has been used to observe cerebral white matter perfusion, but it does not give truly quantitative measures of CBF. WMI showed high WM intensity in magnetic resonance imaging (MRI) T2‐weighted sequence or FLAIR sequence, and low point 40 in T1‐weighted image relative to normal WM and gray matter. However, neither MRI nor FLAIR sequence can accurately reflect the integrity of WM fiber bundles, axon diameter, fiber density, and myelin formation. Diffusion tensor imaging (DTI) can detect magnitude and directionality of water molecules in living tissues. It can display the structure of corticospinal tract through three‐dimensional images. It is more sensitive than conventional MRI and DWI in evaluating the structural integrity of white matter fiber tract. DTI has been widely used in the study of white matter injury under conditions of hemorrhage, ischemia, degeneration, tumors, etc. The injury of white matter was assessed by fractional anisotropy (FA) when using DTI. FA is a common index that comprehensively reflects the integrity of the dimensional bundle, including dimensional density, axon diameter, and white matter sheath shape. The FA value ranges from 0 to 1, 0 indicates the most serious injury, and 1 shows no injury correspondingly. Studies have proved that the decrease in FA value associated with motor function. Both FA and ex vivo DTI showed WMI after ICH. Yang J et al found that the FA value of ipsilateral corpus callosum in baseline state was 0.42 ± 0.02 after ICH accompanied by FA value decreased significantly at days 3, 7, and 28, and the same trend was observed in the capsula interna.

Compared with MRI, the compression of corticospinal tract after basal ganglia ICH can be obtained clearly by DTI. Nowadays, in the surgical treatment of ICH, especially stereotactic hematoma aspiration therapy combined with DTI can identify the extent of WM fiber involvement, avoiding the neurological function aggravation caused by surgery, and thus improve the.

There are many studies on imaging in white matter injury after stroke. More information can be referred to the following literature.^19^, ^37^

## 5 REPAIR OR RECOVERY MECHANISM AND STRATEGY

### 5.1 Self‐repair and recovery

Following an ICH, a few endogenous mechanisms have shown to be involved in the white matter repair and recovery. The majority of studies focus on the clearance of toxic products of hematoma and endogenous neural stem cell migration.

Upregulation of CD36 stimulates macrophages to scavenge extravasated erythrocytes and mediates erythrocyte apoptosis and phagocytosis.^38^ The activation of Nrf2 upregulates the expression of CD163. CD163 is specifically recognized for mediating the creation of hemoglobin‐haptoglobin complexes, which reduces the toxicity of free hemoglobin and enhances cell phagocytosis. CD91, a specific receptor on the cell membrane surface, combines hemopexin‐heme complexes to reduce heme accumulation.^39^ All of them enhance the absorption of the hematoma and alleviate the occupying effect on the white matter.^40^.

There are few studies on the mechanism of ICH‐induced endogenous neuroregeneration. At present, the most popular research is the theory of migration. Subependymal ventricular zone (SVZ) is believed to be the source of oligodendrocyte differentiation and outward migration of neural progenitor cells. Masuda et al reported that the number of BrdU‐positive cells in the subependymal zone and striatum significantly increased in BrdU‐labeled models after intracerebral hemorrhage. By comparing it with the number of BrdU‐positive cells before creating the ICH model, they found that the proliferation of endogenous neural stem cells (NSCs) increased after ICH and new neurons were migrating to the lesion.^41^ There are many endogenous factors influencing the migration of NSCs from the SVZ region (brain‐derived neurotrophic factor, metalloproteinase, and glutamate).^42^, ^43^ However, whether these stem cells involved in nerve regeneration originate solely from the SVZ region has not yet been universally documented, and they may also originate from other places.^44^ Other endogenous nerve regeneration mechanisms need to be noted. Hua et al proved that although thrombin can increase the BBB permeability and cause brain edema and neuronal apoptosis after ICH, it may also improve the neurological function after ICH through nerve and vascular regeneration, but the specific mechanism is still unclear.^45^ Tang et al observed a rat model of ICH for 28 days and found a significant expression of vascular endothelial growth factor (VEGF) and its receptors Flt‐1 and Flk‐1 in the endothelial cells around the hematoma. This natural course stimulating angiogenesis and white matter revascularization may contribute to the white matter self‐repair and recovery.^46^

### 5.2 Neuroprotection

#### 5.2.1 Reducing neuroinflammation

Neuroinflammation is an important pathological mechanism of WMI after ICH. Controlling the inflammatory cascade reaction may not repair the white matter, but it can alleviate the WMI and prevent its aggravation. Previous studies have shown that the central nervous system (CNS) is rich in taurine. After ICH, a large number of amino acids are released or even denatured and inactivated. Supplementing taurine can upregulate the content of H_2_S and reduce neutrophil infiltration and glial activation, downregulate the expression of inflammatory mediators and P2X7R, and reduce the inflammatory response, thereby improving the condition of WMI.^47^ Previous study also demonstrated that endogenous H_2_S reduces NLRP3‐mediated neuroinflammation by inhibiting the P2X7 receptor in rats with ICH.^30^, ^48^ Another study showed that curcumin could inhibit neuroinflammation and alleviate WMI after ICH.^49^ In addition, increasing the expression of procedural death protein 1 and procedural death ligand 1 can induce microglial polarization by regulating neuroimmunity and alleviate inflammatory response after ICH.^50^ After ICH, IL‐17A promotes microglia autophagy and microglia inflammation, while IL‐17A antibody can significantly reduce the brain water content and improve neurological function in mice with ICH. RNA interference with the necessary autophagy genes (ATG5 and ATG7) can inhibit autophagy, reduce microglia autophagy and inflammation, and reduce WMI.^51^ Toll‐like receptor (TLR) antagonists and tumor necrosis factor (TNF) antagonists could alleviate neuroinflammation after ICH by inhibiting HMGB1‐TLR4/NF‐κB‐TNF pathway, thereby protecting the white matter and improving cognitive function.^52^, ^53^, ^54^ Baicalein reduces the levels of inflammatory factors, such as IL‐1β, IL‐4, IL‐6, and TNF‐α, thus controlling the inflammatory response after ICH.^55^ Dimethylamine tetracycline could reduce the iron level in the brain, alleviate neuroinflammation, and consequently reduce brain swelling and neuronal loss, and protect the white matter from secondary damage.^56^

#### 5.2.2 Inhibition of oxidative stress

Duan XC et al have reviewed the treatment strategy for antioxidant stress after ICH.^57^ We mainly reviewed the new treatments in the latest years. Zinc protoporphyrin (ZnPP), an inhibitor of the heme oxygenase, inhibits the degradation of hemoglobin, reduces the contract of carbon monoxide, bilirubin, and iron, and alleviates the WMI induced by ICH.^58^ Baicalein alleviates the effect of oxidative stress via increasing superoxide dismutase (SOD) and glutathione peroxidase (GSH‐Px) activities and downregulating malondialdehyde level.^55^ Intraperitoneal administration of isoliquiritigenin in the acute phase after ICH minimizes brain impairments and neurological deficits, and the mechanisms involved were the regulation of ROS and/or NF‐κB on the activation of NLRP3 inflammasome pathway by the triggering of Nrf2 activity and Nrf2‐induced antioxidant system.^59^

### 5.3 Regenerative repair

#### 5.3.1 Neurogenesis

Shen JF et al found that neurogenesis could be detected in the brain area around the hematoma after ICH.^60^ In the rat model of ICH induced by a collagenase injection, new neurons migrated from the SVZ to the lesion, increasing the regeneration of nerves in the white matter. In addition, an exogenous lactic acid injection after ICH increased the transcription of VEGF and basic fibroblast growth factor (BFGF) through the translocation of nuclear factor‐kappa B (NF‐kappa B), which plays an active role in angiogenesis and neurogenesis.^61^ Otero L et al showed that ICH induced strong endogenous neurogenesis within 72 hours to 7 days after injury, but most of the new cells survived less than 3 weeks due to apoptotic‐mediated apoptosis.^62^ In addition, neurogenesis continued in a small range for at least 1 year after ICH, which provides us with a novel view that promoting endogenous nerve regeneration can improve the prognosis of patients with hemorrhagic stroke.^62^

Another study showed that the clinical neurological function after ICH in mice treated with bone marrow stromal cells was better than that in the control group. The histological results showed that the better outcome was related to the strong activation of endogenous neurogenesis. After transplantation of bone marrow mesenchymal stem cells into the brain of mice, the donor cells subsequently fused into the injured tissues with expression of glial fibrillary acidic protein (GFAP) and neuronal nucleus; thus, this mechanism contributes to the white matter structure repair 63

#### 5.3.2 Oligodendrogenesis

Oligodendrocytes are an important white matter components, accounting for approximately 75% of the subcortical white matter glial cells.^64^ Oligodendrocytes are vulnerable to oxidative stress, excitotoxicity, and damage induced by the apoptotic pathway.^65^, ^66^ There are a few immature oligodendrocytes in the white matter.^67^ Although immature oligodendrocytes, oligodendrocyte precursor cells (OPCs),^68^ have not yet formed a myelin sheath in the white matter, they are gradually developing into mature oligodendrocytes. During the acute stage after ICH, damaged oligodendrocytes cannot produce new myelin sheaths and mature oligodendrocytes cannot proliferate. Therefore, the OPCs stimulated by the lack and injury of the myelin sheath compensate for the proliferation and differentiation of oligodendrocytes by forming new myelin sheaths, which play a key role in the oligodendrocyte‐mediated regeneration and reduce the burden of axonal injury. Michael J et al verified his hypothesis by monitoring the oligodendrocyte lineage in mice model with ICH for 28 days. Myelin was damaged but some myelinated axons survived in the perihematomal area after ICH. The density of oligodendrocyte lineage cells increased as a result of proliferation in the perihematomal region; the number of OPCs and mature oligodendrocytes transiently increased in the perihematomal region, preferentially inside the white matter tracts, and the axons and oligodendrocytes survived for at least 28 days around the hematoma and striatum.^44^ The increase in oligodendrocyte lineage density around the hematoma and striatum after ICH may be due to proliferation, migration from other regions, or both, resulting in oligodendrocyte regeneration.

Insulin‐like growth factor‐1 (IGF‐1) stimulates OPC proliferation in vitro.^69^ Platelet‐derived growth factor (PDGF) increases the ability of proliferation and survival in vitro by activating the α receptor (PDGFRα) on OPCs.^70^, ^71^ Fibroblast growth factor‐2 (FGF‐2) stimulates OPC proliferation^72^ and upregulates the PDGFRα expression in these cells, which prevents them from maturing and, thus, prolongs their responses to PDGF. In line with these findings, thymosin β4 was found to promote oligodendrogenesis by inducing OPCs in a mouse model of ICH.^73^ Nevertheless, there is still no clear research on the migration area of oligodendrocytes after ICH and the effect of the above factors that contribute to the proliferation of oligodendrocytes after human ICH is still unclear. Studies have also shown that microglia cells in vitro and other demyelination in vivo models produce some of the above mediators to promote the proliferation and differentiation of oligodendrocytes.^74^, ^75^, ^76^, ^77^ After stroke, oligodendrocytes are reduced with demyelination and microglia are also activated. Microglia regulate oligodendrocytes by eliminating the damaged cells and the damaged myelin sheaths, thereby facilitating the remyelination. In this process, microglia are transformed from M1 to M2 phenotype (anti‐inflammatory phenotype), which is a classical activation pathway that mediates neuroinflammation.^78^ Microglia with M1 phenotype can kill oligodendrocyte progenitor cells (OPCs) via the TLR4 signaling pathway, while microglia with M2 phenotype have completely different functions. On the theory of relevant experiments in mice with stroke, using the peroxisome proliferator‐activated receptor‐gamma agonist, rosiglitazone, can reduce the M1 phenotype microglia, increase the M2 phenotypes of microglia, induce OPC differentiation, and induce increase in the number of oligodendrocytes.^79^ In case of demyelination, SVZ‐produced OPCs migrated to the damage area of the WM tract (including corpus callosum, fornix fimbria, and striatum) for reformation of the myelin sheath.^80^ It has also been reported in mouse models that neuronal progenitor cells in SVZ generate OPCs and then continue to differentiate and grow into mature myelinated oligodendrocytes to repair the WMI.^81^ Studies in vitro have confirmed the contrasting effects of Inhibin A and Matrilin 2 on OPC differentiation that makes them promising therapeutic targets for white matter recovery after ICH.^82^

#### 5.3.3 Angiogenesis

The stability of both white matter and gray matter depends on the nutrients provided by the blood. ICH can induce angiogenesis and upregulation of the VEGF receptors Flt‐1 and Flk‐1. Increased expression of VEGF, Flt‐1, and Flk‐1 was observed in the cerebral endothelial cells at the hemorrhagic region in the basal ganglia, and the increases of their mRNA persisted up to 28 days.^46^ Regulation of angiogenesis by altering the expression of VEGF and its receptors may be a potential strategy to promote the repair of WMI. GSK126, a novel EZH2 inhibitor, inhibits cell migration and angiogenesis by downregulating VEGF‐A.^83^ Human NSCs promote vascular regeneration, neuroprotection, and functional recovery in mice with stroke by VEGF overexpression.^84^ Chao P et al found that EGb761 attenuates neuronal apoptosis induced by ferrous iron in vitro. Decreased glycogen synthase kinase‐3β activity was observed in EGb761‐treated mice compared with mice with ICH. EGb761 increased the expression of phosphorylated‐GSK3β (Ser9), ribosomal S6 kinase 1, and the Bcl2/Bax ratio to promote angiogenesis.^85^ Repeated exposure to low‐intensity ultrasound increased the expression of collagen and albumin and the extracellular matrix integrity, promoting the formation of a large number of blood vessel‐like structures and capillary vessels around hematomas in rats, and restoring the white matter, thereby improving the neurological function.^86^ Yang et al provided that selective cathepsin B/L inhibitor could promote the vascular regeneration after brain hemorrhage and improve the prognosis of neurological function.^87^ The mechanism may be related to white matter repair, which should be further studied. Tracy J et al have found that oligodendrocyte‐encoded hypoxia‐inducible factors activate the Wnt signaling pathway in the brain to promote postnatal oligodendrocyte precursor cell maturation, and OPCs are an important regulator of angiogenesis. It also shows paracrine secretion activity, induces excessive postpartum leukocyte blood angiogenesis in the body, directly stimulates endothelial cell proliferation in vitro, and promotes blood vessel regenerative and coordinate myelination, ensuring axonal and white matter integrity.^88^ Other experimental and clinical studies indicate that endothelial progenitor cells might mediate endothelial cell regeneration and neovascularization.^89^

#### 5.3.4 Astrogenesis

Subependymal ventricular zone‐generated astrocytes express high levels of thrombospondin (THBS4), a secreted homopentameric glycoprotein. After local cortical ischemic injury, the postnatal SVZ niche increases THBS4 astrocyte production, which then migrate to the damaged cortex. THBS4 activates downstream signals through direct Notch1 receptor binding and endocytosis regulation, including increasing the level of Nfia transcription factor, which is essential for astrocyte production and astrogenesis.^90^ We hypothesized that the destruction of glial cells after ICH would activate this mechanism to stimulate astrogenesis.

Mechanisms of astrogenesis and its role in the restoration of neural function have been reviewed in a specific summary.^91^ However, there are few studies on the effect of astrocytes on the recovery and regeneration after ICH‐induced WMI, which could be a direction of future research.

#### 5.3.5 Axon regeneration

Studies in both human patients with ICH and rat models of ICH show that the invasion of the hematoma into the internal capsule can cause axonal dysfunction,^92^ which would greatly aggravate the severity of symptoms after ICH. The blood‐derived protease thrombin may play a key role in the acute phase of axonal tract injury after ICH in the internal capsule through fragmentation of axonal structures, inducing axonal transport dysfunction.^93^ Therefore, axon regeneration is also a potential treatment.

Myelin‐associated inhibitors can block axonal repair and expansion by binding to the complex receptors of the myelin‐associated inhibitors on the neuronal axons and activating relevant inhibitory signals. For example, chondroitin sulfate proteoglycan (CSPGs) may bind to two myelin‐associated receptor inhibitors (Nogo receptors 1 and 3) that prevent axonal repair and expansion.^94^ Removing the inhibitory effect of CSPG‐rich scar is an important purpose for functional recovery of the CNS after ICH, particularly through overcoming its inhibitory effect on axonal repair. As mentioned in the previous chapter, OPCs proliferate and differentiate in the perihematomal region and have the ability to remyelinate axon tracts after ICH.^44^ Oscar A et al found that hydrogels may act as a physical carrier for cell and axon recombination as well as a scaffold and delivery tool, promoting axonal regeneration after a peripheral nerve lesion in vitro and vivo.^95^ Otero‐Ortega et al reported that adipose‐derived mesenchymal stem cell (ADMSC) therapy can reduce the ischemic lesion volume, restrain cell death, accelerate oligodendrocyte proliferation and myelin formation, and stimulate axon regeneration via paracrine effects, trophic factor production, and immunomodulatory effects.^96^, ^97^ Modulation of melanopsis/GPCR signaling can promote axon regeneration after CNS injury in adults, which may become a target for axon regeneration after ICH.^98^

### 5.4 Stem cell transplantation

Neural stem cells are found in specific regions of the brain of developing and adult mammals, such as the SVZ, the supragranular layers of the cortex, cerebellum, hippocampal dentate gyrus, and the spinal cord. These cells possess a potential to produce populations of neurons and glial lineages. Isolated NSCs can proliferate and differentiate into specific neuronal and glial phenotypes according to different growth factors and culture conditions. We have already mentioned the neural regeneration potential of bone marrow stromal cells and human umbilical cord tissue‐derived cells. In fact, bone marrow and adipose tissue are important sources of bone marrow mesenchymal stem cells, which can differentiate into mesenchymal cells, endodermal cells, and ectodermal cells. Mesenchymal stem cells were significantly stimulated to differentiate into nerve and glial cell line^99^ and could also inhibit the BBB destruction after the brain tissue was generated under appropriate conditions.^100^ Human umbilical cord blood also has the ability to differentiate into glial cells.^101^ Stem cell transplantation as a treatment strategy for WMI—targeting cells instead of neural function, such as protection and regeneration—has a broad application prospect.^102^

As early as in year 2003, Sang‐Wuk Jeong et al by transplanted human NSCs into the intracranial venous of rats in a small rat model of ICH and found that NSCs selectively migrated to the hematoma and differentiated into neurons and stellate cells, aiding white matter repair. There was no difference in the cerebral hemisphere area between the two groups. The mice in the experimental group showed a better limb function than those in the control group after 2 weeks, and they were observed for 8 weeks.^103^ Via a retroviral vector encoding v‐myc, Hong J. Lee et al extracted immortalized cell lines of human NSCs from primary human fetal telencephalon cultures and they detected beta‐galactosidase and mitogen‐activated protein (MAP) 2 in the transplanted NSCs, neurofilaments specific to neurons, and glial fibrillary acidic protein (specific to astrocytes) that could improve the functional outcome around the hematoma in mice.^104^ Wakai T et al have shown that by increasing the expression of SOD and reducing the oxidative stress, the survival rate of NSCs, the number of released paracrine factors, and the number of surviving neurons in the white matter area compressed by the hematoma were increased.^105^ In addition, after ICH, hemoglobin can cause the death of a large number of transplanted NSCs. Hypoxic preconditioning can increase the expression of VEGF secreted by the NSCs and allow adaptation to the toxic effects of hemoglobin around the hematoma.^106^ In the experimental model of a subcortical stroke with WMI, ADMSCs could facilitate the reconstruction of WM fiber tracts and alleviate functional defects.^96^, ^107^

The use of induced pluripotent stem cells (iPS) is the current focus of attention, because these cells can be tailored to patients for self‐use in order to avoid immune rejection, ethical constraints, and tissue donation.^108^ When injected, iPSs mainly differentiate into neurons or glial cells to complete axon synapse regeneration and myelin sheath repair, in order to improve its function after WMI. Moreover, iPSs can be directed to differentiate into characteristic cell types.^109^ For example, iPS cell lines derived from exosome vectors or retroviruses produce a similar number of early neural progenitor cells and glial progenitor cells, while iPS cell lines derived from exosomes produce more OPCs expressing late marker O1 and RIP.^110^ In a related research, iPSs were directed to differentiate into OPCs, which can express myelin‐related proteins to complete remyelination, thus greatly improving the repair of WMI.^111^ In Stewart's experiments, NSC lineages can be differentiated into neuronal lineages that contribute to tissue replacement. In a rat model of a spinal cord WMI, the grafted NSCs provided enough stromal‐derived factor‐1 to improve WM retention and axonal density around the lesion.^112^ NSC transplantation treatment can also reduce the WMI‐induced neuroinflammation. NSCs were transplanted into the lateral ventricles of mice with diffuse white matter lesions. It was found that 2 or 4 weeks after injury, the Sonic hedgehog (Shh) was not upregulated in reactive astrocytes or microglia cells and in SVZ area cells after transplantation of NSCs, and Shh was effectively read mainly in neurons. This can indicate that NSCs had significantly reduced the neuroinflammation after the lesion and could not regulate nerve regeneration through the Shh mechanism.^111^ Piao et al demonstrated an effective source and prospective isolation of human OPCs, which migrated to the WM after transplantation, leading to structural and functional repair.^113^

A latest study reported that Muse cells (multi‐lineage, differentiating stress‐enduring cells), novel nontumorigenic pluripotent stem cells, dwell in the connective tissue and in cultured MSCs and are reported to differentiate into multiple cell types to repair the injured tissue after ICH based on the microenvironment, which may be a tactic of WM repair.^114^

In summary, stem cell transplantation for WMI can promote axonal repair and myelin regeneration, limit the expansion of neuroinflammation, and promote functional recovery with scar formation. Moreover, using stem cell therapy, negative effects, such as immune response, can be avoided. It can be beneficial to the functional recovery of ICH‐induced WMI and thus offer a promising new therapeutic strategy for the complete cure of WMI.

### 5.5 Molecular therapy

#### 5.5.1 Potential microRNA therapy

MicroRNAs (miRNAs) play a key role in the process of inflammation after ICH. There are few studies in terms of the treatment of WMI associated with miRNAs after ICH. Most studies still focus on the control of miRNA‐induced inflammation after ICH, and we have also mentioned the inflammatory injury as a mechanism of WMI after ICH. It is therefore reasonable to speculate that miRNAs can help repair the white matter by reducing the inflammatory response and the edema effect after ICH.

Intracerebral hemorrhage downregulates the expression of miR‐367 and upregulates the expression of IL‐1 receptor (IL‐1R)‐associated kinase (IRAK4) in the primary microglia. miR‐367 controlled the expression of IRAK4 by directly binding to its 3'‐untranslated region, thus restraining NF‐ĸB activation and downstream proinflammatory mediator production. Therefore, based on such inhibition of proinflammatory cytokines, miR‐367 could reduce brain edema and improve brain function.^115^ Zhang Y et al found that miR‐132 could decrease the counts of activated microglia and the expression of proinflammatory cytokines and relieve inflammatory damage by utilizing a unilateral stereotaxic injection to deliver lentiviruses encoding miR‐132, anti‐miR‐132, or an empty lentiviral vector into the caudate nuclei of mice.^116^ Kong et al demonstrated that miRNA‐126 could upregulate the expression of VEGF‐A and downregulate the expression of caspase‐3 to promote angiogenesis after ICH.^117^ miR‐27a‐3p could attenuate the ICH‐induced leukocyte infiltration, reduce the vascular permeability, and upregulate the expression of aquaporin‐11 in the perihematomal area and brain microvascular endothelial cells, thereby inhibiting neuronal apoptosis and microglia activation in the WM area.^118^ miRNA‐223 inhibited inflammation through feedback regulation of NLRP3, an inflammatory corpuscle, thus alleviating brain injury after ICH.^119^ miR‐27b inhibitor diminished iron‐induced oxidative stress, inflammation, and apoptosis by promoting Nrf2/ARE pathway activation^120^; therefore, it may be used for clinical treatment. Another research has shown that upregulation of miR‐129‐5P may alleviate neuroinflammation by inhibiting the HMGB1‐RAGE signaling pathway and thus improve the neurological function in rats with ICH.^121^ David et al found that cofilin siRNA increased the ICH‐induced DNA breakdown, BBB destruction and microglia activation, LPS‐induced NO release, iNOS, COX2, and TNF‐alpha expression, and suppressed oxidative stress in mice. The effect of cofilin knockout on the reduction in 8‐hydroxyguanosine level indicated that injury induced by oxidative stress was reduced.^122^

#### 5.5.2 Gene therapy

Before transplantation, Muse cells can survive in brain tissue accompanied by ICH through gene introduction and cytokine treatment, and can induce different nerve pedigree cells without inducing them into neuronal cell, thereby restoring neural functions.^114^ Knocking down IRAK4 helped restore the function after ICH by significantly inhibiting the NF‐ĸB activation and the downstream production of proinflammatory mediators.^115^ CD47 is a member of the immunoglobulin superfamily, which is mainly expressed on the surface of macrophages, dendrites, and nerves. It can be negatively regulated by interaction with the signal regulatory protein alpha (an inhibitory receptor on the surface of macrophages). The CD47 knockout mice absorbed hematomas more significantly than those in the normal group and with less lesions in the brain tissues.^123^ In TLR4 gene knockout mice with ICH, they found increased expression of microglia and CD36 due to the characteristic of CD36 to induce a strong erythrocyte phagocytosis and therefore accelerate hematoma absorption.^124^

In general, there are few studies on gene repair of white matter after ICH. Yang T et al summarized the gene expression profile after ICH,^125^ providing a potential gene therapy approach.

#### 5.5.3 Nanotechnology

Nanotechnology has been widely used in the monitoring, diagnosis, prevention, damage repair, and treatment, particularly in the aspect of cancer in biological systems. Although nanotechnology is rarely used in the recovery of WMI after ICH, we reviewed the previous literatures and acquired the application prospects of nanotechnology in the repair of ICH.

An in vivo experiment has proved that peptide‐based nanofiber scaffold could provide an admitted environment for axon regeneration and accelerate wound healing as well as that RADA16‐I scaffold could reduce the inflammation and apoptosis, and promote long‐term functional recovery in rats after ICH.^126^ RGD‐containing elastin‐like polypeptide (REP) has high affinity for cells with nonimmune cytotoxicity and inflammatory responses. In the acute phase of ICH in rats, REP can reduce the hematoma volume, the count of activated microglia, and the expression of von Willebrand factor (vWF) and can also prevent the leakage of immunoglobulin G (IgG) into the brain parenchyma without blocking the microvessels.^127^ Based on the characteristics of nanotechnology, we hypothesize that future studies will focus on the repair of WM after ICH, although such studies are relatively rare.

### 5.6 Clinical treatment

Currently widely used clinical treatment methods include etiological screening (imaging), early hematoma clearance, monitoring treatment (vital signs, blood glucose, electrolytes, intracranial pressure, laboratory parameters, and CT review), preventive treatment (rebleeding, edema occupying position, seizures, pneumonia, and intracranial infections), neuroprotective agents, hypothermia, and early rehabilitation.^128^ Unfortunately, whether these treatments play an important role in the recovery of white matter after ICH remains unclear. Further investigations are required to directly address these issues. There are few studies combining basic and clinical findings. However, we believe that an inherent white matter repair mechanism exists, providing inspiration for a subsequent research.

## 6 SUMMARY

The white matter is involved in the transmission of motor and sensory information between the cerebral cortex, subcortical structures, and spinal cord. Therefore, in spite of bleeding or ischemic stroke, WMI can cause severe cognitive dysfunction, mood disorders, and motor dysfunction. Without the parallel protection of white matter, true lasting neurorestoration cannot be achieved.

Although some advances have been made in ICH study, many questions remain to be addressed. First, studies on pathophysiological mechanisms of WMI after ICH should be continued, while adequate attention should be payed on the repair or recovery mechanism of white matter after ICH. Second, translation of regimen proven to be potential in basic researches into clinical treatments should be strengthened in the future, considering mice are the species different from human. Third, the vast majority of basic researches regarding WMI after ICH focuses on single‐factor treatment, which is insufficient. Multimodal therapies are needed to be tested in further studies.
